# A genetically immortalized human stem cell line: a promising new tool for Alzheimer's disease therapy

**DOI:** 10.17179/excli2015-560

**Published:** 2015-10-21

**Authors:** Nicha Puangmalai, Alyma Somani, Wipawan Thangnipon, Clive Ballard, Martin Broadstock

**Affiliations:** 1 King’s College London, Wolfson Centre for Age-Related Diseases, London, SE1 1UL, UK; 2 Research Center for Neuroscience, Institute of Molecular Biosciences, Mahidol University, Salaya, Nakhonpathom 73170, Thailand

**Keywords:** Alzheimer's disease, cell viability, CTX0E03 cell line, neural stem cell, okadaic acid, oligomeric amyloid-ß

## Abstract

Amyloid-β peptides and hyper-phosphorylated tau are the main pathological hallmarks of Alzheimer's disease (AD). Given the recent failure of several large-scale clinical trials and the lack of disease-modifying pharmacological treatments, there is an urgent need to develop alternative therapies. A clinical grade human CTX0E03 neural stem cell line has recently passed phase I trials in people with stroke. However, this cell line has not been investigated in other neurodegenerative disorders. This study investigates the survival of CTX0E03 cells under conditions based on the underlying AD pathology. Cell viability assays showed a concentration dependence of this cell line to the toxic effects of Aβ_1-42_, but not Aβ_1-40_, and okadaic acid, a phosphatase 2A inhibitor. Notably, CTX0E03 cell line displayed toxicity at concentrations significantly higher than both rat neural stem cells and those previously reported for primary cultures. These results suggest CTX0E03 cells could be developed for clinical trials in AD patients.

## Introduction

Dementia affects over 35 million people worldwide, and this is set to rise to 115 million by 2050 (Broadstock et al., 2014[6]). This devastating condition incurs enormous personal cost to those affected, with an estimated worldwide financial cost of $ 604 billion (Wimo et al., 2013[51]). Alzheimer's disease (AD) is the most common cause of dementia, affecting up to two-thirds of people with the condition (Cornutiu, 2015[12]). The progressive nature of the cognitive decline in people with AD leads to the need for complex treatment and care. Thus, AD represents a major and increasing public health concern and there is an urgent imperative to develop more effective therapies both to treat and to delay the onset of disease. 

Major pathological hallmarks of AD are the deposition of aggregated amyloid β-protein (Aβ) contributing to the formation of extracellular amyloid plaques (senile plaques) and the intra-neuronal accumulation of hyper-phosphorylated tau protein, which, over time, lead to synaptic dysfunction, neuronal loss and neuroinflammation (Hardy and Selkoe, 2002[21]; Reitz and Mayeux, 2014[42]). Cleavage of amyloid precursor protein (APP) by β- and γ-secretase to generate soluble Aβ species that form additional Aβ plaques can induce neurotoxicity and synaptic failure (Haass and Selkoe, 2007[20]). Aβ_1-40_ and Aβ_1-42_ are two prevalent forms that show a high tendency to initiate congregation into soluble forms and eventually to form insoluble aggregated fibrils as amyloid plaques (Lui and Zhao 2004[34]). 

In the UK, there are four licensed pharmacological treatments only for AD, viz., donepezil, galantamine and rivastigmine, acetylcholinesterase inhibitors, which are prescribed for mild to moderate AD; and the NMDA receptor antagonist memantine that is licensed for moderate to severe AD (Rafii and Aisen, 2015[41]). Whilst these treatments provide symptomatic benefit and are cost-effective, their benefits extend for an average of only 6-24 months, and none targets the underlying AD pathology and disease processes (Bond et al., 2012[5]). Although these treatments are enormously valuable in treating symptoms, there is an urgent need to develop better, more effective treatments designed to ameliorate the disease progression (Broadstock et al., 2014[6]).

The recent failures of several large-scale phase III trials aimed at reducing the amyloid burden in AD have emphasized the necessity to broaden treatment approaches (Doody et al., 2013[16], 2014[17]; Salloway et al., 2014[43]). One potential treatment that has generated considerable excitement is stem cell implantation. Some successes have been reported with cellular therapies in a number of other neurodegenerative diseases, such as Parkinson's disease, and a number of transgenic animal studies point to this as a potentially exciting therapeutic option in the treatment of AD (Chen and Blurton-Jones, 2012[9]; Blurton-Jones et al., 2014[4]; Oh et al., 2014[37]; Buttery and Barker, 2014[8]). There are, however, a number of challenges to overcome before studies in AD patients can be considered. Firstly, viable candidate human grade cell have to be available and capable of surviving in the toxic AD brain environment, as Aβ species have a reproducible toxic impact on cultured cortical neurons and correlations have been reported between the severity of Aβ plaques and reduction in endogenous neurogenesis (Chuang, 2010[11]; Mu and Gage, 2011[36]). Secondly, the abnormally hyper-phosphorylated tau protein in AD brain aggregates into neurofibrillary tangles in neurons correlating with the blockage of intracellular trafficking of neurotrophins and other functional proteins (Wang et al., 2014[50]). However, the roles of tau phosphorylation in neurogenesis still are not clear, with studies reporting beneficial effects of tau hyper-phosphorylation on an adult stem cell neurogenesis, but others showing tau phosphorylation not participating in the proliferation of neural precursors (Schindowski et al., 2008[45]; Hong et al., 2011[24]). 

A human grade neural stem cell line, CTX0E03, has recently been developed (Pollock et al., 2006[40]). This cell line is a clonal human cortical multipotent stem cell line created by genetic modification of first trimester human fetal cortical cells with a single-copy integration of *c-*mycER^TAM^ fusion gene. Assays for cell line identity (molecular assays using qRT-PCR, telomerase assay, southern blotting, karyotyping and cellular phenotype using immunocytochemistry) have been developed and a number of safety aspects of the c-mycER technology have been confirmed *in vitro* to rule out the possibility of reactivation of cells by re-exposure to 4-OHT, tamoxifen or endogenous steroid hormones (Pollock and Sinden, 2008[39]). In addition, the cell line has shown to confer benefit in rodent studies of stroke (Mack, 2011[35]; Smith et al., 2012[46]; Hassani et al., 2012[22]; Hicks et al., 2013[23]). A phase Ia clinical trial in people with stroke has shown good tolerability with encouraging indications of benefit (PISCES trial, NCT01151124), and a phase II study is about to commence. 

In order to assess whether the CTX cell line affords long term benefit to AD patients, the key question is the tolerance of these cells to Aβ and hyper-phosphorylated tau. Thus this study investigated the resistance of CTX0E03 cells to the toxicity of oligomeric amyloid-β species and to okadaic acid, a phosphatase inhibitor.

## Materials and Methods

### CTX0E03 cell culture

CTX0E03 cells were obtained from ReNeuron (UK). Derivation, culturing, and characterization of the cells were as previously described (Pollock et al., 2006[40]). CTX0E03 cells were routinely cultured at 37 °C under humidified atmosphere of 5 % CO_2_ on mouse laminin-I (20 μg/ml, PathClear) freshly coated flask in Dulbecco's modified Eagle's medium nutrient F-12 HAM mixture (Sigma) containing 10 ng/ml basic fibroblast growth factor (PeproTech), 20 ng/ml epidermal growth factor (PeproTech), 100 nM 4-OHT (Sigma), 200 μM L-ascorbic acid (Sigma), 2 mM Glutamax supplement (Gibco), and 1X N-2 supplement (Gibco). At 70-90 % confluence, cells were passaged using Accutase solution (Sigma) for 2-5 min at 37 °C and sedimented at 1000 *g* for 5 min. Cells were reseeded for expansion in supplemented media for experimental use. All experiments were conducted using CTX0E03 cells between passages 33 to 36.

### Neural stem cell culture

Rat fetal neural stem cells (NSCs) (Invitrogen), isolated from cortices of fetal (embryonic day 14) Spraque-Dawley rats, were expanded on 0.01 % poly-L-ornithine solution (Sigma)-coated flask in KnockOut^TM^ DMEM/F12 medium supplemented with 20 ng/ml EGF, 20 ng/ml bFGF, StemPro^®^ NSC SFM supplement, and 2 mM Glutamax (all from Invitrogen). Cells were passaged and reseeded as described above when cells reached 80-90 % confluency. Rat fetal NSCs used in the study were passaged not more than 3 times.

### Amyloid preparation 

Oligomeric Aβ_1-40_ and Aβ_1-42_ peptides (California Peptide Research) were prepared as previously described (Dahlgren et al., 2002[13]). In briefly, peptides were dissolved in hexafluoro-2-propanol (Sigma), divided into smaller aliquots, dried and the peptide films stored at -80 °C. Soluble peptides were prepared by diluting peptide films in anhydrous dimethylsulfoxide (DMSO) (Sigma) to 5 mM, then further diluted to 100 μM in phosphate-buffered saline (PBS), and incubated at 4 °C for 24 h before adding to cells. 

### Okadaic acid preparation

Okadaic acid (OA) (Abcam) was dissolved in DMSO, diluted to desired concentrations, and stored at 4 °C until used.

### Cell treatment

CTX0E03 cells or rat fetal NSCs were seeded onto precoated 96-well plates at 50,000 cells/ml. Cells were treated with Aβ_1-40_ and Aβ_1-42_ at 0.5, 1, 5, 10, and 15 μM, or with OA at 0.5, 1, 5, 10, and 15 nM. Vehicle control cultures were treated with PBS or DMSO (for Aβ and OA respectively). Cells were incubated at 37 °C for 24 h prior to cell viability assay.

### Cell viability assay

Cells were incubated with PrestoBlue (Invitrogen) for 30 min at 37 °C, and cell viability then was measured in a FlexStation 3 microplate reader at an excitation of 535 nm and emission of 615 nm. 

### Lactate dehydrogenase (LDH)-cytotoxicity assay

LDH-cytotoxicity assay was conducted according to the manufacturer's protocol (Abcam) (Lai et al., 2014[31]). In short, culture medium of treated cells was transferred into a 96-well plate containing substrate mixture and after 30 min incubation at room temperature, LDH activity was measured at A_450 nm_ in a FlexStation 3 microplate reader, with reference wavelength of 650 nm.

### Statistical analysis

All assays were conducted in three individual experiments in triplicate and reported as mean ± SD. Statistical analysis was evaluated using One-way analysis of variance (ANOVA), followed by Bonferroni Post-Hoc analysis using Graph-Pad Prism software (Graph Pad Software version 5.0a, USA). Statistical significance is accepted when *p* *< *0.05.

## Results

### Effects of oligomeric amyloid β-peptides on CTX0E03 cells

**Figure 1:**
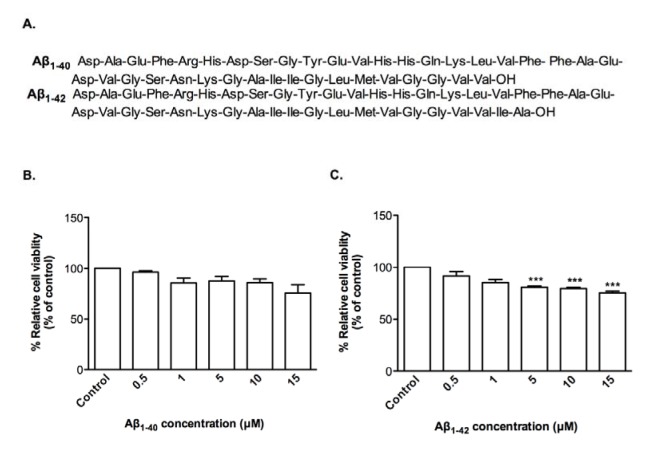
Effects of oligomeric Aβ peptides on CTX0E03 cell viability. Primary structure of Aβ peptides used (A). Cells were incubated for 24 h in the presence of indicated concentrations of oligomeric Aβ_1-40_ (B) and Aβ_1-42 _(C). Cell viability was measured using PrestoBlue reagent. Controls contained vehicle. Results are reported as mean ± SD of three individual experiments performed in triplicate. Statistical analysis was evaluated using ANOVA followed by Bonferroni Post-Hoc analysis. ***p < 0.001 compared to control

In order to investigate whether clinical grade CTX0E03 cells were affected by oligomeric Aβ_1-40_ and Aβ_1-42_ (Figure 1A(Fig. 1)), cell viability assays were performed, which do not show any significant effect of Aβ_1-40_ relative to vehicle control (Figure 1B(Fig. 1)), but there is significantly decrease in cell viability with Aβ_1-42_ over the range of 5 to 15 μM re-sulting to 80.9 %, 79.8 % and 75.4 % relative to control (50 % effective concentration (EC_50_) = 36.43 μM) (Figure 1C(Fig. 1)).

**Figure 2:**
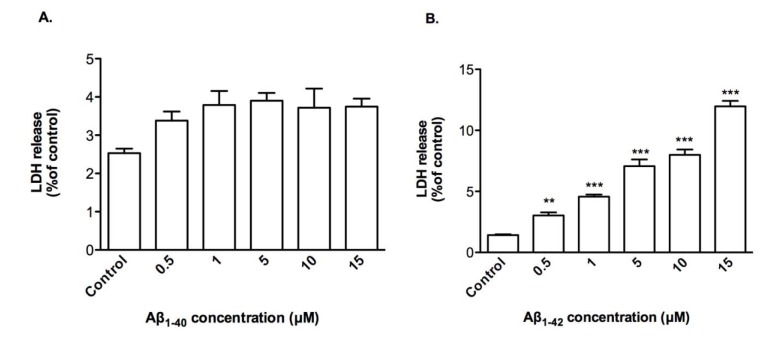
Effects of oligomeric Aβ peptides on CTX0E03 cell permeability. Cells were incubated for 24 h in the presence of indicated concentrations of oligomeric Aβ_1-40_ (A) and Aβ_1-42_ (B) prior to performing LDH assay. Results are reported as mean ± SD of three individual experiments performed in triplicate. Statistical analysis was evaluated using ANOVA followed by Bonferroni Post-Hoc analysis. **p < 0.01, ***p < 0.001 compared to control

Cytoxicity by Aβ_1-42_ was due to membrane damage as demonstrated by LDH release modestly by up to 12.0 % from treated CTX0E03 cells with 15 μM of Aβ_1-42_ (Figure 2B(Fig. 2)) and, as expected, this was not observed with of Aβ_1-40_ treatment (Figure 2A(Fig. 2)).

### Effects of OA on CTX0E03 cells

**Figure 3:**
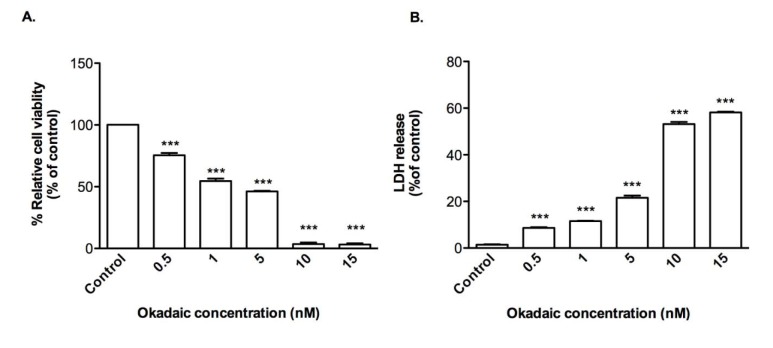
Effects of okadaic acid on CTX0E03 cell viability and permeability. Cells were incubated for 24 h in the presence of indicated concentrations of okadaic acid and cell viability (A) and permeability (B) was measured using PrestoBlue reagent and LDH assay respectively. Results are reported as mean ± SD of three individual experiments performed in triplicate. Statistical analysis was evaluated using ANOVA followed by Bonferroni Post-Hoc analysis. ***p < 0.001 compared with control

OA is a protein phosphatase inhibitor used to induce tau hyper-phosphorylation. Treatment of CTX0E03 cells with OA resulted in loss of cell viability (EC_50_ = 1.66 nM) (Figure 3A(Fig. 3)) and release of LDH by reaching to 8.6 %, 11.6 %, 21.6 %, 53.2 %, and 58.2 % over the range of 0.5-15 nM of OA exposure (Figure 3B(Fig. 3)), but at a 1,000-fold lower concentration than that required using Aβ_1-42_.

Effects of oligomeric amyloid β-peptides and OA on neural stem cells

**Figure 4:**
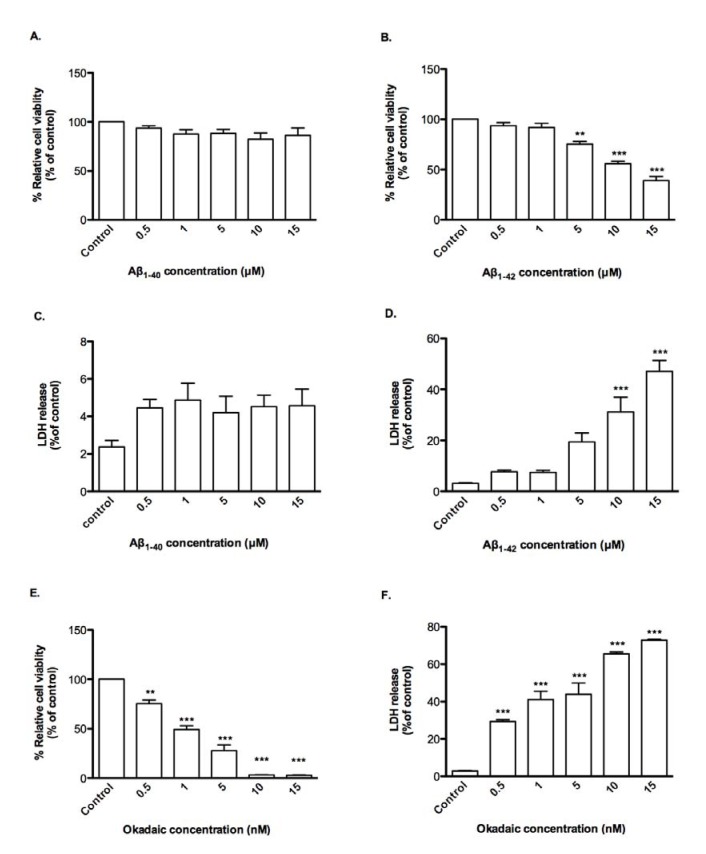
Effects of oligomeric Aβ peptides and okadaic acid on NSC viability and permeability. Cells were incubated for 24 h in the presence of indicated concentrations of oligomeric Aβ peptides or okadaic acid and cell viability (A, B, E) and permeability (C, D, F) was measured using PrestoBlue reagent and LDH assay respectively. Results are reported as mean ± SD of three individual experiments performed in triplicate. Statistical analysis was evaluated using ANOVA followed by Bonferroni Post-Hoc analysis. **p < 0.01, ***p < 0.001 compared to control

The susceptibilities of CTX0E03 cells to Aβ peptides and OA were compared with those of rat fetal NSCs., the latter being more sensitive were more sensitive, viz., viability in presence of Aβ_1-42_ and OA had EC_50_ of 11.89 μM and 1.20 nM respectively and LDH release in a concentration-dependent manner compared with control (Figure 4(Fig. 4)). Again, Aβ_1-40_ was inactive over the concentration range tested.

## Discussion

We demonstrate that Aβ_1-42_, but not Aβ_1-40_, at μM range were toxic to clinical grade CTX0E03 stem cells over a 24-h exposure. Rat fetal NSCs were more sensitive. Both cell lines were 1,000-fold more sensitive to OA toxicity under the same experimental settings.

*In vitro* neurotoxicity studies have reported that oligomeric Aβ species are substantially more toxic (EC_50 _≤ 10 μM) to neuronal cortical cultures than either protofibrils or fibrils (EC_50 _> 10 μM) (Ahmed et al., 2010[1]; Kitiyanant et al., 2012[28]; Dai et al., 2013[14]). The underlying mechanism of Aβ oligomer toxicity to cortical neurons includes induction of Ca^2+^ entry through NMDA and AMPA receptors, causing mitochondrial Ca^2+^ overload, subsequent oxidative stress and mitochondrial membrane depolarization (Butterfield et al., 2007[7], Alberdi et al., 2010[2]). Western blotting has revealed a sequential up-regulation of Bim (within 24 h) and down-regulation of Bcl-2 (after 48 h) leading to activation of pro-apoptotic factors including Bax and subsequent neuronal cell death (Kudo et al., 2012[30]). Moreover, 10 μM Aβ_1-42 _treatment of rat cortical cultures 24 h also causes nearly 50 % apoptotic cell death, manifested by increases in apoptotic protein expression, including increased activated caspase-3 and elevation in level of reactive oxygen species (Thangnipon et al., 2012[49], 2013[48]; Suwanna et al., 2014[47]). Aβ is also toxic to various cell lines, e.g., PC12 cell line (Feng et al., 2013[19]). In addition, there is an association between the amount of Aβ oligomers in cerebrospinal fluid and cognitive decline (Santos et al., 2012[44]), and specific forms of oligomers are associated with synaptic and cognitive dysfunction (Lesne et al., 2006[32]; Ono et al., 2009[38]). 

CTX0E03 cell line is relatively tolerant to the *in vitro* effects of different species of oligomeric Aβ. However, Aβ levels in post-mortem AD brain are in pM to nM range (Eucher et al., 2007[18]). That Aβ_1-40_ was nontoxic (≤ 15 µm for 24 h) to CTX0E03 cells and rat fetal NSCs are in agreement with a previous study showing that Aβ_1-40_ does not interfere with cell survival of mouse neural stem cells (Itokazu et al., 2014[26]). Hicks et al. (2013[23]) have demonstrated that CTX0E03 cells express both trophic and pro-angiogenic factors (VEGF, EGF, bFGF, ANGPT1, TGFβ1, HIF-1α and ANGPT2). 

In AD, tau is abnormally hyper-phosphorylated and forms into paired helical filaments in neurons (Iqbal et al., 2010[25]). Activation of protein kinase and/or inhibition of protein phosphatases are the likely cause(s) of tau hyper-phosphorylation (Wang et al., 2014[50]). OA, a phosphatase 2A inhibitor, has been used widely to induce hyper-phosphorylation of tau in order to stimulate neural death in culture and enhance Aβ deposition, synaptic loss, as well as memory impairment in AD animal models (Barrio et al., 2011[3]; Li et al., 2011[33]; Kamat et al., 2013[27]). CTX0E03 cells were exquisitely susceptible to OA cytotoxicity. DMSO as vehicle does not affect CTX and NS cell viability compared to culture media or PBS (data not shown) (Kong et al., 2015[29]). OA treatment in primary rat cortical neurons resulted in an increased number of mature lysosomes and induced neuronal cell death (Cho et al., 2013[10]). High concentrations of OA decrease axonal outgrowth and branching, as well as inducing depression of growth-associated protein-43 (an axonal growth regulator) (Das and Miller, 2012[15]). 

In conclusion, our study indicates the ability of clinical grade CTX0E03 stem cell line to resist the inhospitable milieu associated with AD. Thus, CTX0E03 stem cells provide a potential candidate for cell therapy in AD patients.

## Acknowledgements

This research project is supported by Mahidol University and Thailand Research Fund (IRG5780009) and the Thailand Research Fund (TRF) Royal Golden Jubilee Ph.D. scholarship (Grant no. PHD/0175/ 2552). MB and CB acknowledge the support of the National Institute for Health Research (NIHR) Mental Health Biomedical Research Centre and Dementia Unit at South London and Maudsley NHS Foundation Trust and Institute of Psychiatry King’s College London. This article presents independent research partially supported by the National Institute for Health Research (NIHR). The views expressed are those of the authors and are not necessarily those of NHS, NIHR or Department of Health. MB is funded by the Edmond and Lily Safra Research Foundation. The authors are grateful to Prof. Prapon Wilairat, Mahidol University, for critical reading and valuable suggestions that improved the manuscript.
